# Scoliosis epidemiology is not the same all over the world: a study from a scoliosis school screening in the island of Chongming, China

**DOI:** 10.1186/1748-7161-9-S1-O43

**Published:** 2014-12-04

**Authors:** Qing Du, Stefano Negrini, Xuan Zhou, Xiaohua He, Jianan Li, Li Zhao, Peijie Chen

**Affiliations:** 1 Department of Rehabilitation of Xin Hua Hospital Affiliated to Shanghai Jiao Tong University School of Medicine, Shanghai, China; 2 Department of Clinical and Experimental Sciences of University of Brescia, Brescia, Italy; 3 Palmer College of Chiropractic, Florida, USA; 4 Rehabilitation Department of Jiangsu Province Hospital, Jiangsu Province, China; 5 Department of Pediatric Orthopedic of Xin Hua Hospital Affiliated to Shanghai Jiao Tong University School of Medicine, Shanghai, China; 6 Department of Kinesiology of Shanghai University of Sport, Shanghai, China

**Keywords:** School scoliosis screening, scoliosis, prevalence, China

## Background

Genetic factors in China could be different than in other places and reflect in prevalence differences.

## Aim

The aim of the present study was to exam if some scoliotic parameters, such as patients’ age, gender ratio, curve magnitude, curve type, and curve side, in Chongming Island with few population exchanges with remaining China differed from that of the published date through a scoliosis school screening program.

## Design

Cross-sectional study.

## Methods

A total of 6824 children (3477 boys and 3347girls) aged from 6 to 17 years old recruited from 352 classes of primary schools, and junior and senior high schools were screened for scoliosis. 442 children showed physical signs of potential scoliosis (Angle of Trunk Rotation at Adam’s forward bending test of five degrees or more) and were referred for posteroanterior radiographic evaluation. Radiographic evaluation included the Cobb angle, curve type, and curve side of scoliosis. The differences in the prevalence rate, the distribution of curve parameters and the variables of age and gender were analyzed by SPSS.

## Results

The prevalence rate of scoliosis (> 10 degrees or more) was 2.52% (172 of 6824 schoolchildren). There was a positive but very weak correlation between scoliosis and age. The prevalence rate was significantly higher in girls than in boys (girls vs. boys: 3.11% vs. 1.96%, ratio 1.5:1). Most of the curves were minor (from 10 to 19 degrees). The right curve was the most common type in the thoracic region (60.3% of all thoracic curves), while it was left in thoracolumbar (75.5%) and lumbar regions (64.7%).

## Conclusions

The prevalence of scoliosis in the Island of Chongming was 2.52%. The percentage of curve magnitude and type were comparable, while gender, curve side and the correlation between scoliosis and age in the Island of Chongming differed from that in other countries. According to these results, epidemiological regional variability, possibly with genetic basis, can be considered.

